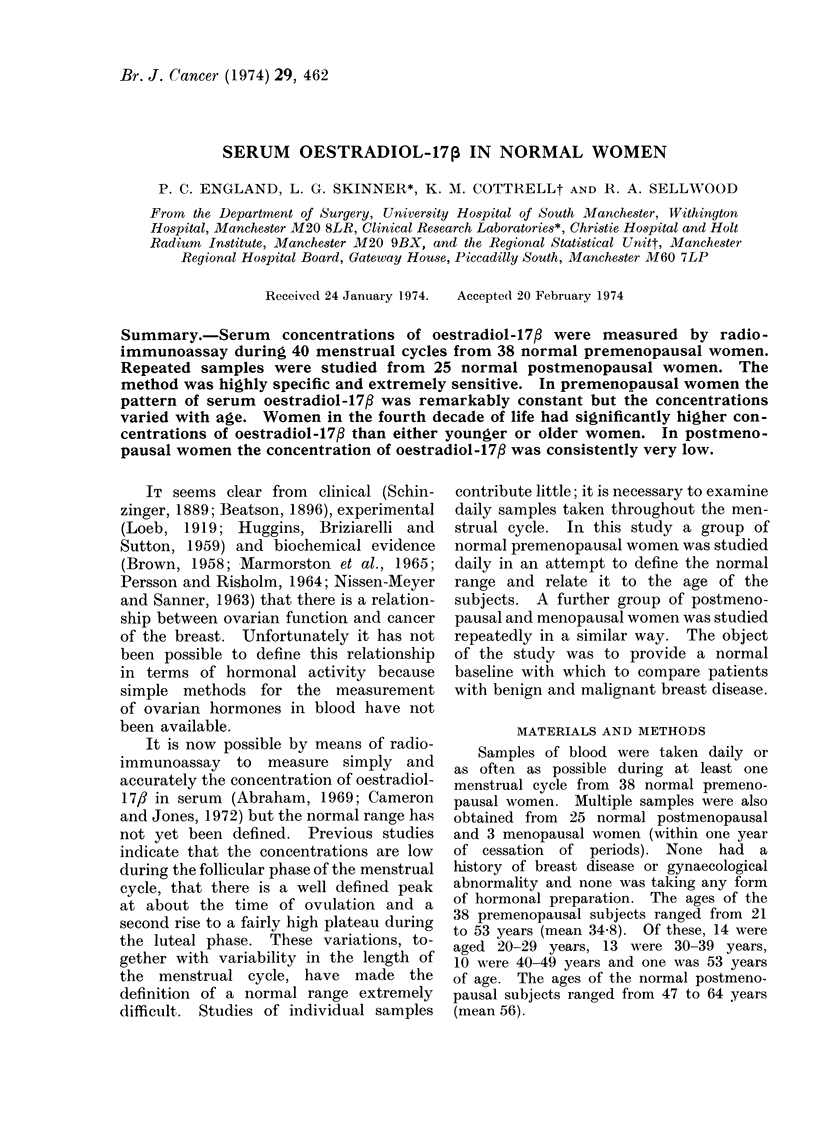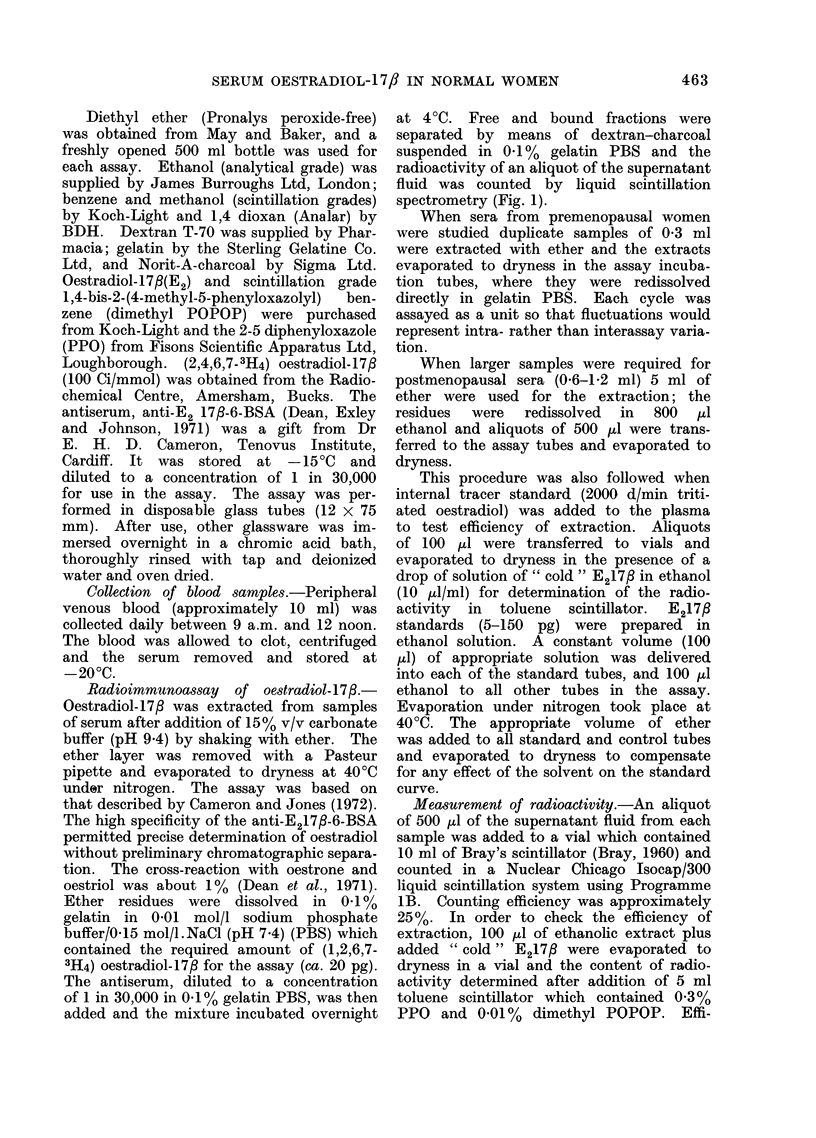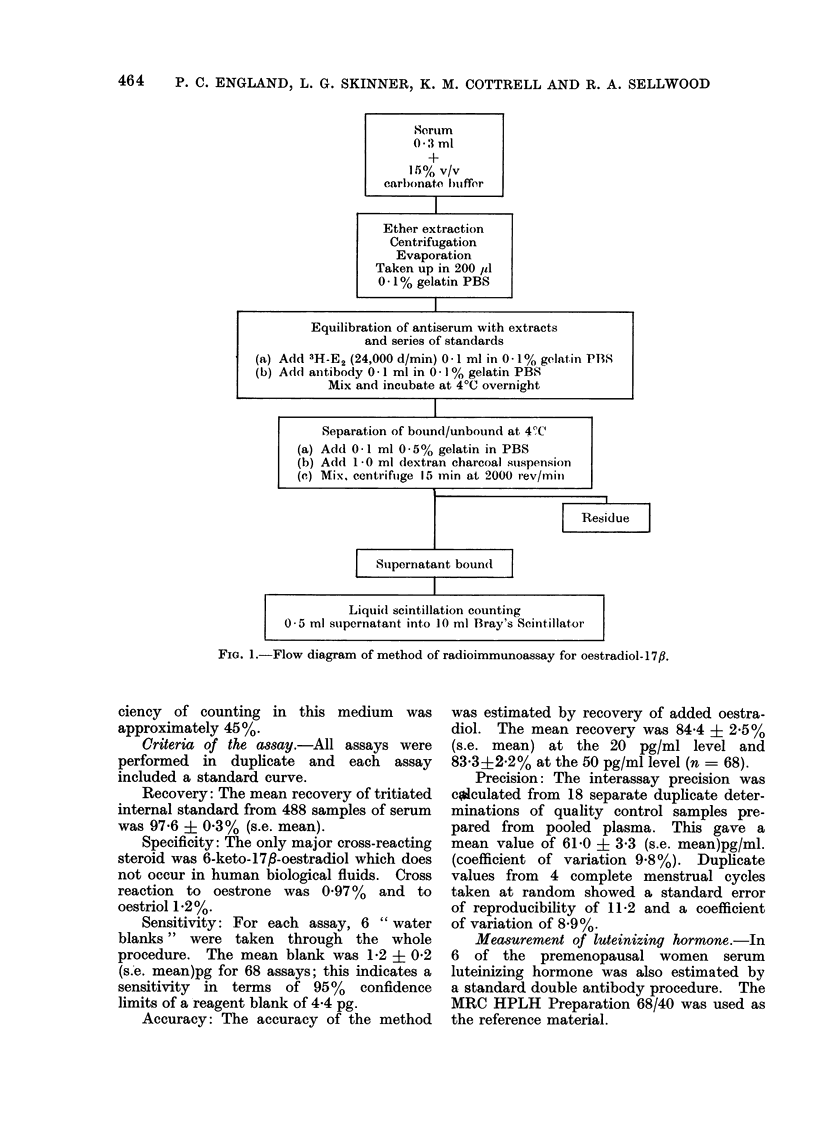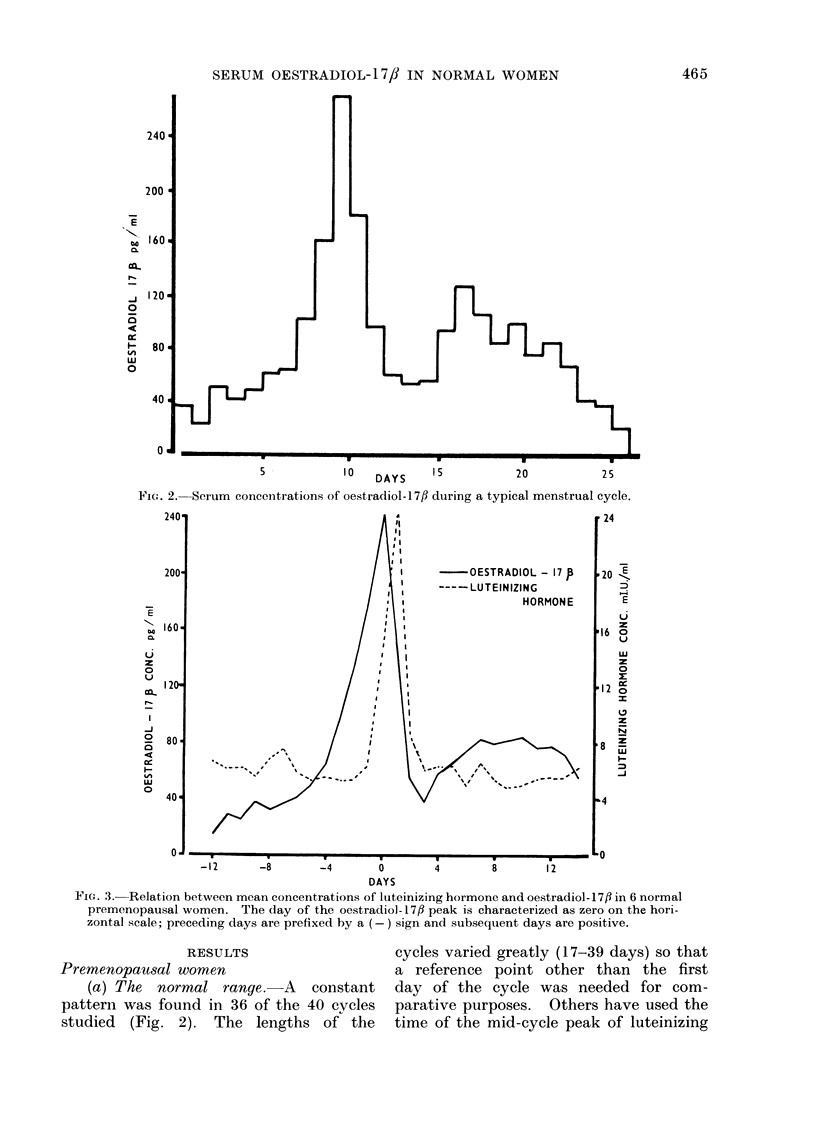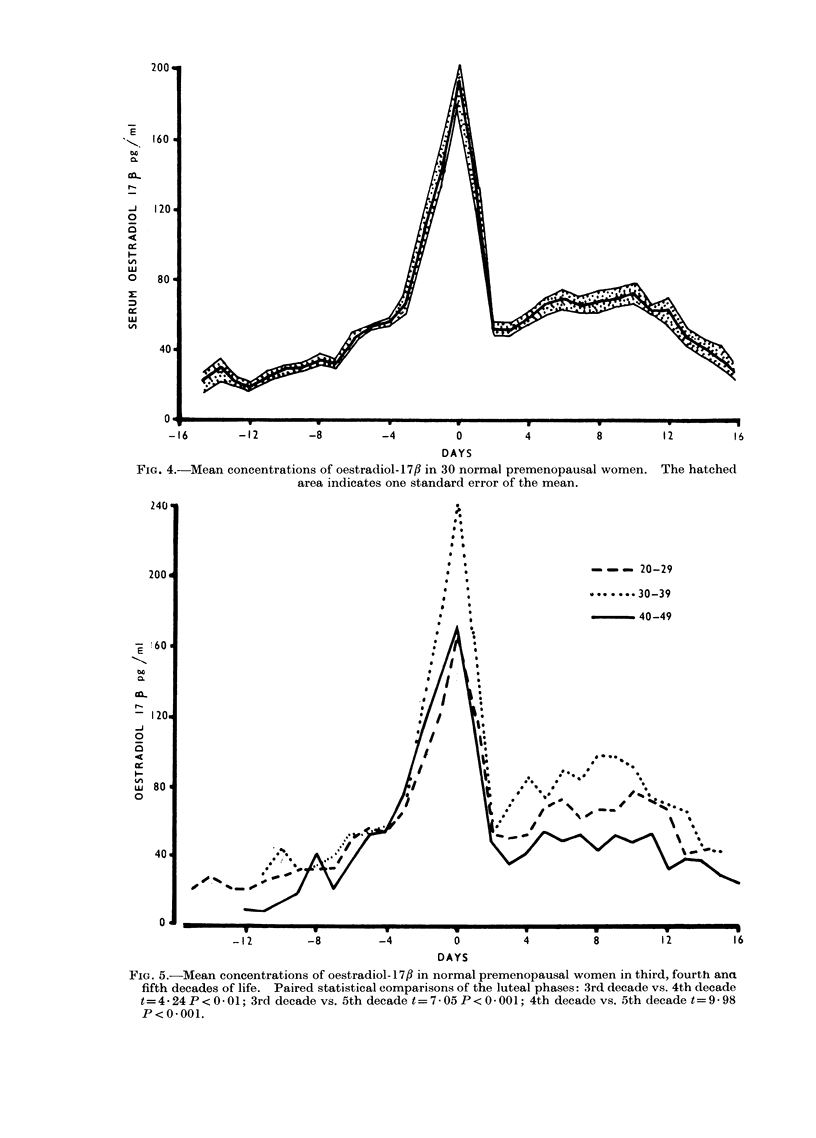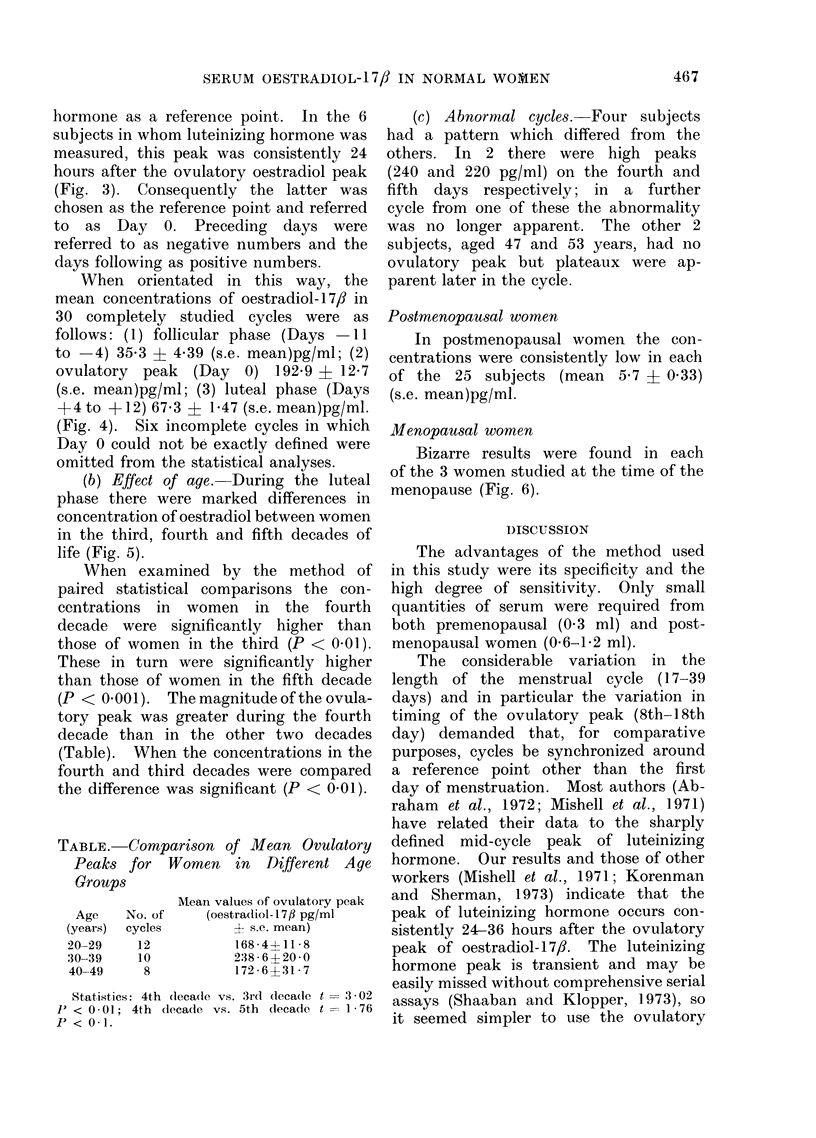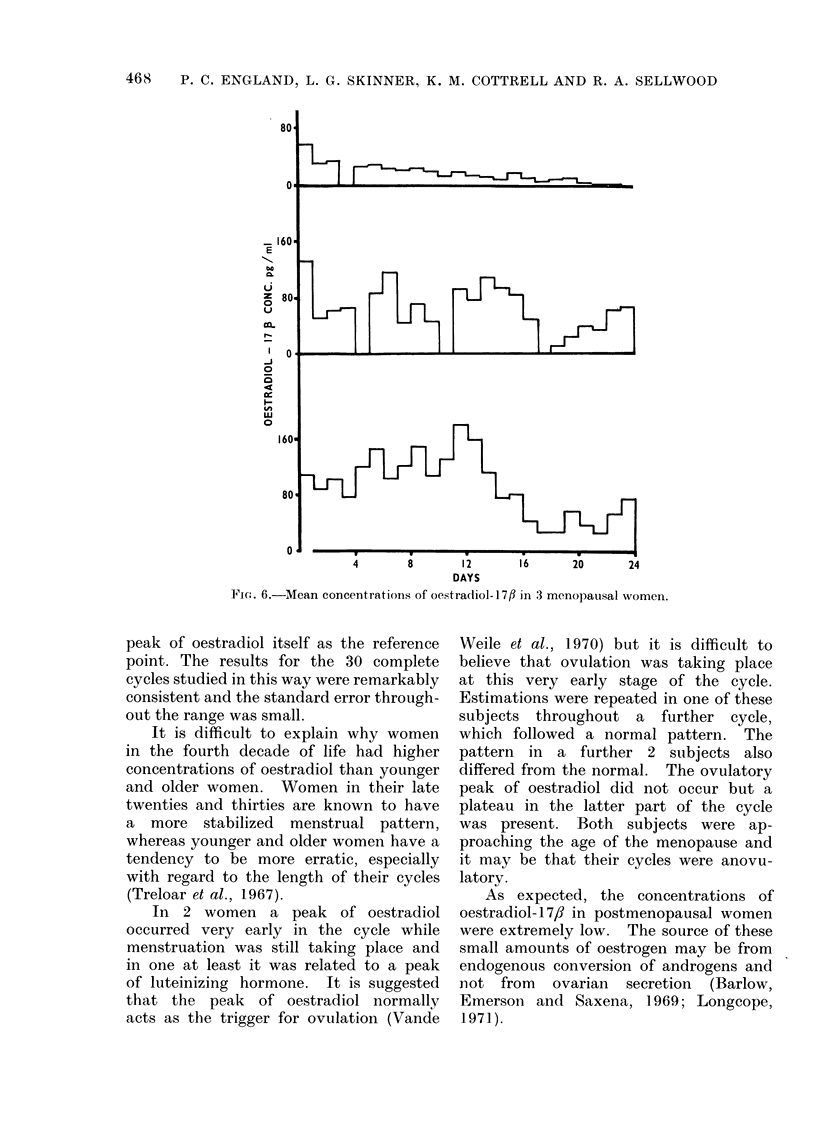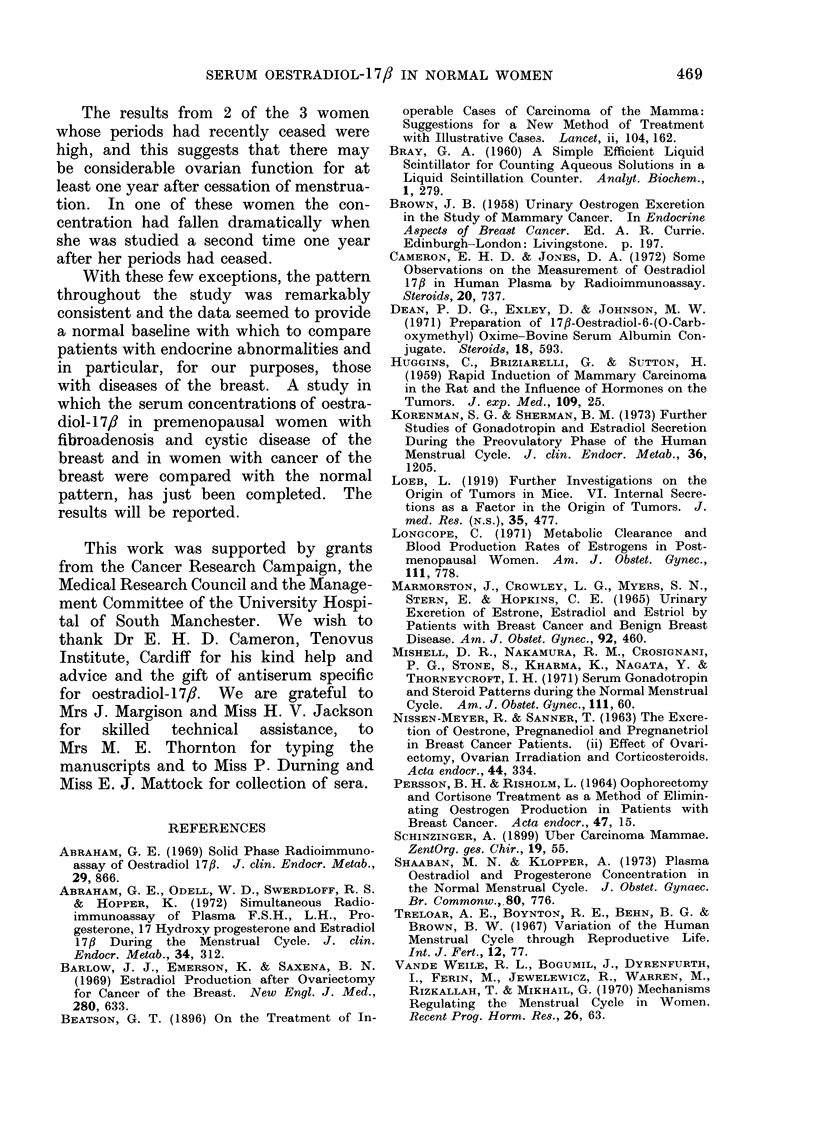# Serum Oestradiol-17β in Normal Women

**DOI:** 10.1038/bjc.1974.98

**Published:** 1974-06

**Authors:** P. C. England, L. G. Skinner, K. M. Cottrell, R. A. Sellwood

## Abstract

Serum concentrations of oestradiol-17β were measured by radio-immunoassay during 40 menstrual cycles from 38 normal premenopausal women. Repeated samples were studied from 25 normal postmenopausal women. The method was highly specific and extremely sensitive. In premenopausal women the pattern of serum oestradiol-17β was remarkably constant but the concentrations varied with age. Women in the fourth decade of life had significantly higher concentrations of oestradiol-17β than either younger or older women. In postmenopausal women the concentration of oestradiol-17β was consistently very low.


*Br. J. Cancer* (1974) **29**, 462

### SERUM OESTRADIOL-17β IN NORMAL WOMEN

P. C. ENGLAND, L. G. SKINNER*, K. M. COTTRELL† AND R. A. SELLWOOD

*From the Department of Surgery, University Hospital of South Manchester, Withington Hospital, Manchester M20 8LR, Clinical Research Laboratories*, Christie Hospital and Holt Radium Institute, Manchester M20 9BX, and the Regional Statistical Unit†, Manchester Regional Hospital Board, Gateway House, Piccadilly South, Manchester M60 7LP*

Received 24 January 1974. Accepted 20 February 1974

**Summary.—Serum concentrations of oestradiol-17β were measured by radioimmunoassay during 40 menstrual cycles from 38 normal premenopausal women. Repeated samples were studied from 25 normal postmenopausal women. The method was highly specific and extremely sensitive. In premenopausal women the pattern of serum oestradiol-17β was remarkably constant but the concentrations varied with age. Women in the fourth decade of life had significantly higher concentrations of oestradiol-17β than either younger or older women. In postmenopausal women the concentration of oestradiol-17β was consistently very low.**

It seems clear from clinical (Schinzinger, 1889; Beatson, 1896), experimental (Loeb, 1919; Huggins, Briziarelli and Sutton, 1959) and biochemical evidence (Brown, 1958; Marmorston *et al.*, 1965; Persson and Risholm, 1964; Nissen-Meyer and Sanner, 1963) that there is a relationship between ovarian function and cancer of the breast. Unfortunately it has not been possible to define this relationship in terms of hormonal activity because simple methods for the measurement of ovarian hormones in blood have not been available.

It is now possible by means of radioimmunoassay to measure simply and accurately the concentration of oestradiol-17β in serum (Abraham, 1969; Cameron and Jones, 1972) but the normal range has not yet been defined. Previous studies indicate that the concentrations are low during the follicular phase of the menstrual cycle, that there is a well defined peak at about the time of ovulation and a second rise to a fairly high plateau during the luteal phase. These variations, together with variability in the length of the menstrual cycle, have made the definition of a normal range extremely difficult. Studies of individual samples contribute little; it is necessary to examine daily samples taken throughout the menstrual cycle. In this study a group of normal premenopausal women was studied daily in an attempt to define the normal range and relate it to the age of the subjects. A further group of postmenopausal and menopausal women was studied repeatedly in a similar way. The object of the study was to provide a normal baseline with which to compare patients with benign and malignant breast disease.

### MATERIALS AND METHODS

Samples of blood were taken daily or as often as possible during at least one menstrual cycle from 38 normal premenopausal women. Multiple samples were also obtained from 25 normal postmenopausal and 3 menopausal women (within one year of cessation of periods). None had a history of breast disease or gynaecological abnormality and none was taking any form of hormonal preparation. The ages of the 38 premenopausal subjects ranged from 21 to 53 years (mean 34·8). Of these, 14 were aged 20–29 years, 13 were 30–39 years, 10 were 40–49 years and one was 53 years of age. The ages of the normal postmenopausal subjects ranged from 47 to 64 years (mean 56).

Diethyl ether (Pronalys peroxide-free) was obtained from May and Baker, and a freshly opened 500 ml bottle was used for each assay. Ethanol (analytical grade) was supplied by James Burroughs Ltd, London; benzene and methanol (scintillation grades) by Koch-Light and 1,4 dioxan (Analar) by BDH. Dextran T-70 was supplied by Pharmacia; gelatin by the Sterling Gelatine Co. Ltd, and Norit-A-charcoal by Sigma Ltd. Oestradiol-17$\beta$($E_2$) and scintillation grade 1,4-bis-2-(4-methyl-5-phenyloxazolyl) benzene (dimethyl POPOP) were purchased from Koch-Light and the 2-5 diphenyloxazole (PPO) from Fisons Scientific Apparatus Ltd, Loughborough. (2,4,6,7-$^3H_4$) oestradiol-17$\beta$ (100 Ci/mmol) was obtained from the Radiochemical Centre, Amersham, Bucks. The antiserum, anti-$E_2$ 17$\beta$-6-BSA (Dean, Exley and Johnson, 1971) was a gift from Dr E. H. D. Cameron, Tenovus Institute, Cardiff. It was stored at $-15$°C and diluted to a concentration of 1 in 30,000 for use in the assay. The assay was performed in disposable glass tubes ($12 \times 75$ mm). After use, other glassware was immersed overnight in a chromic acid bath, thoroughly rinsed with tap and deionized water and oven dried.

*Collection of blood samples.*—Peripheral venous blood (approximately 10 ml) was collected daily between 9 a.m. and 12 noon. The blood was allowed to clot, centrifuged and the serum removed and stored at $-20$°C.

*Radioimmunoassay of oestradiol*-17$\beta$.—Oestradiol-17$\beta$ was extracted from samples of serum after addition of 15% v/v carbonate buffer (pH 9·4) by shaking with ether. The ether layer was removed with a Pasteur pipette and evaporated to dryness at 40°C under nitrogen. The assay was based on that described by Cameron and Jones (1972). The high specificity of the anti-$E_2$17$\beta$-6-BSA permitted precise determination of oestradiol without preliminary chromatographic separation. The cross-reaction with oestrone and oestriol was about 1% (Dean *et al.*, 1971). Ether residues were dissolved in 0·1% gelatin in 0·01 mol/l sodium phosphate buffer/0·15 mol/l.NaCl (pH 7·4) (PBS) which contained the required amount of (1,2,6,7-$^3H_4$) oestradiol-17$\beta$ for the assay (*ca.* 20 pg). The antiserum, diluted to a concentration of 1 in 30,000 in 0·1% gelatin PBS, was then added and the mixture incubated overnight at 4°C. Free and bound fractions were separated by means of dextran–charcoal suspended in 0·1% gelatin PBS and the radioactivity of an aliquot of the supernatant fluid was counted by liquid scintillation spectrometry (Fig. 1).

When sera from premenopausal women were studied duplicate samples of 0·3 ml were extracted with ether and the extracts evaporated to dryness in the assay incubation tubes, where they were redissolved directly in gelatin PBS. Each cycle was assayed as a unit so that fluctuations would represent intra- rather than interassay variation.

When larger samples were required for postmenopausal sera (0·6–1·2 ml) 5 ml of ether were used for the extraction; the residues were redissolved in 800 $\mu$l ethanol and aliquots of 500 $\mu$l were transferred to the assay tubes and evaporated to dryness.

This procedure was also followed when internal tracer standard (2000 d/min tritiated oestradiol) was added to the plasma to test efficiency of extraction. Aliquots of 100 $\mu$l were transferred to vials and evaporated to dryness in the presence of a drop of solution of " cold " $E_2$17$\beta$ in ethanol (10 $\mu$l/ml) for determination of the radioactivity in toluene scintillator. $E_2$17$\beta$ standards (5–150 pg) were prepared in ethanol solution. A constant volume (100 $\mu$l) of appropriate solution was delivered into each of the standard tubes, and 100 $\mu$l ethanol to all other tubes in the assay. Evaporation under nitrogen took place at 40°C. The appropriate volume of ether was added to all standard and control tubes and evaporated to dryness to compensate for any effect of the solvent on the standard curve.

*Measurement of radioactivity.*—An aliquot of 500 $\mu$l of the supernatant fluid from each sample was added to a vial which contained 10 ml of Bray's scintillator (Bray, 1960) and counted in a Nuclear Chicago Isocap/300 liquid scintillation system using Programme 1B. Counting efficiency was approximately 25%. In order to check the efficiency of extraction, 100 $\mu$l of ethanolic extract plus added " cold " $E_2$17$\beta$ were evaporated to dryness in a vial and the content of radioactivity determined after addition of 5 ml toluene scintillator which contained 0·3% PPO and 0·01% dimethyl POPOP. Effi-

FIG. 1.—Flow diagram of method of radioimmunoassay for oestradiol-17$\beta$.

ciency of counting in this medium was approximately 45%.

*Criteria of the assay.*—All assays were performed in duplicate and each assay included a standard curve.

Recovery: The mean recovery of tritiated internal standard from 488 samples of serum was 97·6 ± 0·3% (s.e. mean).

Specificity: The only major cross-reacting steroid was 6-keto-17$\beta$-oestradiol which does not occur in human biological fluids. Cross reaction to oestrone was 0·97% and to oestriol 1·2%.

Sensitivity: For each assay, 6 " water blanks " were taken through the whole procedure. The mean blank was 1·2 ± 0·2 (s.e. mean)pg for 68 assays; this indicates a sensitivity in terms of 95% confidence limits of a reagent blank of 4·4 pg.

Accuracy: The accuracy of the method was estimated by recovery of added oestradiol. The mean recovery was 84·4 ± 2·5% (s.e. mean) at the 20 pg/ml level and 83·3±2·2% at the 50 pg/ml level ($n$ = 68).

Precision: The interassay precision was calculated from 18 separate duplicate determinations of quality control samples prepared from pooled plasma. This gave a mean value of 61·0 ± 3·3 (s.e. mean)pg/ml. (coefficient of variation 9·8%). Duplicate values from 4 complete menstrual cycles taken at random showed a standard error of reproducibility of 11·2 and a coefficient of variation of 8·9%.

*Measurement of luteinizing hormone.*—In 6 of the premenopausal women serum luteinizing hormone was also estimated by a standard double antibody procedure. The MRC HPLH Preparation 68/40 was used as the reference material.

FIG. 2.—Serum concentrations of oestradiol-17β during a typical menstrual cycle.

FIG. 3.—Relation between mean concentrations of luteinizing hormone and oestradiol-17β in 6 normal premenopausal women. The day of the oestradiol-17β peak is characterized as zero on the horizontal scale; preceding days are prefixed by a ( — ) sign and subsequent days are positive.

### RESULTS

*Premenopausal women*

(*a*) *The normal range.*—A constant pattern was found in 36 of the 40 cycles studied (Fig. 2). The lengths of the cycles varied greatly (17–39 days) so that a reference point other than the first day of the cycle was needed for comparative purposes. Others have used the time of the mid-cycle peak of luteinizing

FIG. 4.—Mean concentrations of oestradiol-17β in 30 normal premenopausal women. The hatched area indicates one standard error of the mean.

FIG. 5.—Mean concentrations of oestradiol-17β in normal premenopausal women in third, fourth and fifth decades of life. Paired statistical comparisons of the luteal phases: 3rd decade vs. 4th decade $t=4{\cdot}24$ $P<0{\cdot}01$; 3rd decade vs. 5th decade $t=7{\cdot}05$ $P<0{\cdot}001$; 4th decade vs. 5th decade $t=9{\cdot}98$ $P<0{\cdot}001$.

hormone as a reference point. In the 6 subjects in whom luteinizing hormone was measured, this peak was consistently 24 hours after the ovulatory oestradiol peak (Fig. 3). Consequently the latter was chosen as the reference point and referred to as Day 0. Preceding days were referred to as negative numbers and the days following as positive numbers.

When orientated in this way, the mean concentrations of oestradiol-17$\beta$ in 30 completely studied cycles were as follows: (1) follicular phase (Days $-11$ to $-4$) $35{\cdot}3 \pm 4{\cdot}39$ (s.e. mean)pg/ml; (2) ovulatory peak (Day 0) $192{\cdot}9 \pm 12{\cdot}7$ (s.e. mean)pg/ml; (3) luteal phase (Days $+4$ to $+12$) $67{\cdot}3 \pm 1{\cdot}47$ (s.e. mean)pg/ml. (Fig. 4). Six incomplete cycles in which Day 0 could not be exactly defined were omitted from the statistical analyses.

(*b*) *Effect of age*.—During the luteal phase there were marked differences in concentration of oestradiol between women in the third, fourth and fifth decades of life (Fig. 5).

When examined by the method of paired statistical comparisons the concentrations in women in the fourth decade were significantly higher than those of women in the third ($P < 0{\cdot}01$). These in turn were significantly higher than those of women in the fifth decade ($P < 0{\cdot}001$). The magnitude of the ovulatory peak was greater during the fourth decade than in the other two decades (Table). When the concentrations in the fourth and third decades were compared the difference was significant ($P < 0{\cdot}01$).

TABLE.—*Comparison of Mean Ovulatory Peaks for Women in Different Age Groups*

| Age (years) | No. of cycles | Mean values of ovulatory peak (oestradiol-17$\beta$ pg/ml ± s.e. mean) |
|---|---|---|
| 20–29 | 12 | 168·4±11·8 |
| 30–39 | 10 | 238·6±20·0 |
| 40–49 | 8 | 172·6±31·7 |

Statistics: 4th decade vs. 3rd decade $t = 3{\cdot}02$ $P < 0{\cdot}01$; 4th decade vs. 5th decade $t = 1{\cdot}76$ $P < 0{\cdot}1$.

(*c*) *Abnormal cycles*.—Four subjects had a pattern which differed from the others. In 2 there were high peaks (240 and 220 pg/ml) on the fourth and fifth days respectively; in a further cycle from one of these the abnormality was no longer apparent. The other 2 subjects, aged 47 and 53 years, had no ovulatory peak but plateaux were apparent later in the cycle.

*Postmenopausal women*

In postmenopausal women the concentrations were consistently low in each of the 25 subjects (mean $5{\cdot}7 \pm 0{\cdot}33$) (s.e. mean)pg/ml.

*Menopausal women*

Bizarre results were found in each of the 3 women studied at the time of the menopause (Fig. 6).

### DISCUSSION

The advantages of the method used in this study were its specificity and the high degree of sensitivity. Only small quantities of serum were required from both premenopausal (0·3 ml) and postmenopausal women (0·6–1·2 ml).

The considerable variation in the length of the menstrual cycle (17–39 days) and in particular the variation in timing of the ovulatory peak (8th–18th day) demanded that, for comparative purposes, cycles be synchronized around a reference point other than the first day of menstruation. Most authors (Abraham *et al*., 1972; Mishell *et al*., 1971) have related their data to the sharply defined mid-cycle peak of luteinizing hormone. Our results and those of other workers (Mishell *et al*., 1971; Korenman and Sherman, 1973) indicate that the peak of luteinizing hormone occurs consistently 24–36 hours after the ovulatory peak of oestradiol-17$\beta$. The luteinizing hormone peak is transient and may be easily missed without comprehensive serial assays (Shaaban and Klopper, 1973), so it seemed simpler to use the ovulatory

FIG. 6.—Mean concentrations of oestradiol-17$\beta$ in 3 menopausal women.

peak of oestradiol itself as the reference point. The results for the 30 complete cycles studied in this way were remarkably consistent and the standard error throughout the range was small.

It is difficult to explain why women in the fourth decade of life had higher concentrations of oestradiol than younger and older women. Women in their late twenties and thirties are known to have a more stabilized menstrual pattern, whereas younger and older women have a tendency to be more erratic, especially with regard to the length of their cycles (Treloar *et al.*, 1967).

In 2 women a peak of oestradiol occurred very early in the cycle while menstruation was still taking place and in one at least it was related to a peak of luteinizing hormone. It is suggested that the peak of oestradiol normally acts as the trigger for ovulation (Vande Weile *et al.*, 1970) but it is difficult to believe that ovulation was taking place at this very early stage of the cycle. Estimations were repeated in one of these subjects throughout a further cycle, which followed a normal pattern. The pattern in a further 2 subjects also differed from the normal. The ovulatory peak of oestradiol did not occur but a plateau in the latter part of the cycle was present. Both subjects were approaching the age of the menopause and it may be that their cycles were anovulatory.

As expected, the concentrations of oestradiol-17$\beta$ in postmenopausal women were extremely low. The source of these small amounts of oestrogen may be from endogenous conversion of androgens and not from ovarian secretion (Barlow, Emerson and Saxena, 1969; Longcope, 1971).

The results from 2 of the 3 women whose periods had recently ceased were high, and this suggests that there may be considerable ovarian function for at least one year after cessation of menstruation. In one of these women the concentration had fallen dramatically when she was studied a second time one year after her periods had ceased.

With these few exceptions, the pattern throughout the study was remarkably consistent and the data seemed to provide a normal baseline with which to compare patients with endocrine abnormalities and in particular, for our purposes, those with diseases of the breast. A study in which the serum concentrations of oestradiol-17β in premenopausal women with fibroadenosis and cystic disease of the breast and in women with cancer of the breast were compared with the normal pattern, has just been completed. The results will be reported.

This work was supported by grants from the Cancer Research Campaign, the Medical Research Council and the Management Committee of the University Hospital of South Manchester. We wish to thank Dr E. H. D. Cameron, Tenovus Institute, Cardiff for his kind help and advice and the gift of antiserum specific for oestradiol-17β. We are grateful to Mrs J. Margison and Miss H. V. Jackson for skilled technical assistance, to Mrs M. E. Thornton for typing the manuscripts and to Miss P. Durning and Miss E. J. Mattock for collection of sera.

### REFERENCES

ABRAHAM, G. E. (1969) Solid Phase Radioimmunoassay of Oestradiol 17β. *J. clin. Endocr. Metab.*, **29**, 866.

ABRAHAM, G. E., ODELL, W. D., SWERDLOFF, R. S. & HOPPER, K. (1972) Simultaneous Radioimmunoassay of Plasma F.S.H., L.H., Progesterone, 17 Hydroxy progesterone and Estradiol 17β During the Menstrual Cycle. *J. clin. Endocr. Metab.*, **34**, 312.

BARLOW, J. J., EMERSON, K. & SAXENA, B. N. (1969) Estradiol Production after Ovariectomy for Cancer of the Breast. *New Engl. J. Med.*, **280**, 633.

BEATSON, G. T. (1896) On the Treatment of Inoperable Cases of Carcinoma of the Mamma: Suggestions for a New Method of Treatment with Illustrative Cases. *Lancet*, ii, 104, 162.

BRAY, G. A. (1960) A Simple Efficient Liquid Scintillator for Counting Aqueous Solutions in a Liquid Scintillation Counter. *Analyt. Biochem.*, **1**, 279.

BROWN, J. B. (1958) Urinary Oestrogen Excretion in the Study of Mammary Cancer. In *Endocrine Aspects of Breast Cancer*. Ed. A. R. Currie. Edinburgh–London: Livingstone. p. 197.

CAMERON, E. H. D. & JONES, D. A. (1972) Some Observations on the Measurement of Oestradiol 17β in Human Plasma by Radioimmunoassay. *Steroids*, **20**, 737.

DEAN, P. D. G., EXLEY, D. & JOHNSON, M. W. (1971) Preparation of 17β-Oestradiol-6-(O-Carboxymethyl) Oxime–Bovine Serum Albumin Conjugate. *Steroids*, **18**, 593.

HUGGINS, C., BRIZIARELLI, G. & SUTTON, H. (1959) Rapid Induction of Mammary Carcinoma in the Rat and the Influence of Hormones on the Tumors. *J. exp. Med.*, **109**, 25.

KORENMAN, S. G. & SHERMAN, B. M. (1973) Further Studies of Gonadotropin and Estradiol Secretion During the Preovulatory Phase of the Human Menstrual Cycle. *J. clin. Endocr. Metab.*, **36**, 1205.

LOEB, L. (1919) Further Investigations on the Origin of Tumors in Mice. VI. Internal Secretions as a Factor in the Origin of Tumors. *J. med. Res.* (N.S.), **35**, 477.

LONGCOPE, C. (1971) Metabolic Clearance and Blood Production Rates of Estrogens in Postmenopausal Women. *Am. J. Obstet. Gynec.*, **111**, 778.

MARMORSTON, J., CROWLEY, L. G., MYERS, S. N., STERN, E. & HOPKINS, C. E. (1965) Urinary Excretion of Estrone, Estradiol and Estriol by Patients with Breast Cancer and Benign Breast Disease. *Am. J. Obstet. Gynec.*, **92**, 460.

MISHELL, D. R., NAKAMURA, R. M., CROSIGNANI, P. G., STONE, S., KHARMA, K., NAGATA, Y. & THORNEYCROFT, I. H. (1971) Serum Gonadotropin and Steroid Patterns during the Normal Menstrual Cycle. *Am. J. Obstet. Gynec.*, **111**, 60.

NISSEN-MEYER, R. & SANNER, T. (1963) The Excretion of Oestrone, Pregnanediol and Pregnanetriol in Breast Cancer Patients. (ii) Effect of Ovariectomy, Ovarian Irradiation and Corticosteroids. *Acta endocr.*, **44**, 334.

PERSSON, B. H. & RISHOLM, L. (1964) Oophorectomy and Cortisone Treatment as a Method of Eliminating Oestrogen Production in Patients with Breast Cancer. *Acta endocr.*, **47**, 15.

SCHINZINGER, A. (1899) Uber Carcinoma Mammae. *ZentOrg. ges. Chir.*, **19**, 55.

SHAABAN, M. N. & KLOPPER, A. (1973) Plasma Oestradiol and Progesterone Concentration in the Normal Menstrual Cycle. *J. Obstet. Gynaec. Br. Commonw.*, **80**, 776.

TRELOAR, A. E., BOYNTON, R. E., BEHN, B. G. & BROWN, B. W. (1967) Variation of the Human Menstrual Cycle through Reproductive Life. *Int. J. Fert.*, **12**, 77.

VANDE WEILE, R. L., BOGUMIL, J., DYRENFURTH, I., FERIN, M., JEWELEWICZ, R., WARREN, M., RIZKALLAH, T. & MIKHAIL, G. (1970) Mechanisms Regulating the Menstrual Cycle in Women. *Recent Prog. Horm. Res.*, **26**, 63.